# Presbyopia: a New Potential Pharmacological Treatment

**Published:** 2012

**Authors:** Jorge Benozzi, Giovanna Benozzi, Betina Orman

**Affiliations:** 1 Fundación Argentina de Glaucoma, Buenos Aires, Argentina; 2 Pharmacological Unit, Faculty of Dentistry, Buenos Aires University, Argentina

**Keywords:** Presbyopia, Pharmacological treatment, Accommodation

## Abstract

Presbyopia occurs after 40 years of age in humans with a progressive loss of accommodation. Accommodation depends on the contraction of the ciliary muscle and iris, lens changes and convergence. The parasympathetic system regulates the degree of ciliary muscle and iris contraction necessary to modify the shape and position of the lens and its stimulation is effective through the activation of muscarinic receptors that are present in both structures. The hypothesis proposed here suggests the correction of accommodation in emmetropic presbyopic patients using a pharmacological treatment that includes a cholinergic agent combined with non-steroidal anti-inflammatory drugs (NSAIDs). This drug combination can restore near vision without affecting distance vision. It is important to note that the pharmaceutical form used was devoid of any inflammatory or other collateral effects.

## INTRODUCTION

Presbyopia is the progressive loss of accommodation resulting in loss of the visual ability to focus on objects located at different distances. Accommodation in humans is performed by ciliary muscle and iris sphincter contractions, convergence and changes in the shape and position of the lens [1,2]. The latter action is passive, meaning that the lens changes are dependent on the ciliary muscle and iris contractions. Also, when the centre of the accommodation is active, the ciliary muscle contraction is stimulated and miosis and convergence occurs in normal binocular patients [2, 3]. 

The iris and ciliary muscle have muscarinic receptors that are stimulated by the parasympathetic system through its cholinergic neurotransmitter acetylcholine [4]. This stimulation produces contraction of the ciliary muscle and changes in pupil size by changing the shape and position of the lens, and consequently provokes accommodative capacity related to the parasympathetic activity [5-7]. As presbyopia has been corrected from the thirteenth century with glasses, and since the last century with contact lenses and surgery [8,9], a pharmacological treatment to restore accommodation is proposed here.

## HYPOTHESIS

It is well known that when parasympathetic stimulation takes place with pilocarpine, the generation of ciliary muscle spasmodic contraction occurs and lens thickness increases, which in turn increases the focus depth [7,10]. This situation improves near vision but decreases distance vision because the lens cannot change its thickness or position [6,11]. Combining NSAIDs with parasympathetic agonists, the intensity of the contraction of the pupil [12] and the ciliary muscle was decreased, allowing the lens to change shape and position for good vision at all distances.

The combination with NSAIDs eliminates local inflammation that always appears as secondary to the chronic stimulation of pilocarpine (fixed pupil, posterior synechiae and pigment dispersion) [12]. 

In this sense, the hypothesis aims to resolve accommodation in emmetropic presbyopic patients using a pharmacological treatment, as described above, through parasympathetic stimulation combined with NSAIDs.

## DISCUSSION

According to the hypothesis criteria, work has been performed for a decade on muscarinic stimulation with carbachol, pilocarpine and physostigmine, an anticholinesterase inhibitor. In all cases, near vision was restored with more or less success according to the quality and concentration of the stimulating agent. A loss of far and half distance vision in all patients treated was observed [2,11]. However, chronic stimulation with any of these drugs caused an inflammatory reaction of the anterior uvea. When a non-steroidal anti-inflammatory (diclofenac) was added to the parasympathetic agonist pilocarpine the pharmacological combination restored near vision without causing blurred far and half distance vision or inflammatory reactions. If the combination of the parasympathetic agonist with a steroidal anti-inflammatory (dexamethasone) was used, an inflammatory reaction was prevented. It was also observed that near vision was restored, but blurred far and half distance vision was noticed. Therefore, a clinical benefit was observed in the patients treated with a combination of pilocarpine and diclofenac.

The uveal tract chronic stimulation using the parasympathomimetic agent caused inflammatory reactions, pigment dispersion, posterior synechiae and spasmodic contractions of the ciliary muscle and iris, which resulted in a fixed pupil and myopic shift [11,13].

The NSAIDs, which are able to inhibit cyclooxygenase(s) activity, act as anti-inflammatory agents in the anterior uveal tract, decreasing miosis and spasmodic ciliary contractions, pigment dispersion and posterior synechia, because they diminish the local inflammation caused by the chronic instillation of parasympathomimetic drugs [12,14,15].

In this sense, it was found that the combination of pilocarpine with diclofenac was the most effective formula that could be used chronically for the treatment of presbyopia without the occurrence of side-effects (pilocarpine 1% and 0.1% diclofenac, [1% pilocarpina Poen, Poen, Buenos Aires, Argentina. Voltaren 0.1%, Novartis, Buenos Aires, Argentina] at 6 hour intervals during the daily hours). Within the 100 subjects treated, 20 patients presented ocular burning and discomfort right after drop instillation and only one of them abandoned treatment for these cause. The others four patients who deserted pharmacological treatment were fearful to the chronically drop instillation.

Two hundred emmetropic eyes of 100 patients of both sexes with presbyopia, in the age range of 45 years to 50 years, were treated during a 5 year period. All of patients had a near vision of Jaeger 1 (J1) and a far vision of 20/20 with an eye drop instillation at 6 hour interval the daily hours. None had ocular or systemic diseases and 95% of the patients were under treatment. Moreover, 1% of the patients discontinued treatment for ocular burning and discomfort, while 4% preferred treatment with glasses.

**Figure1:**
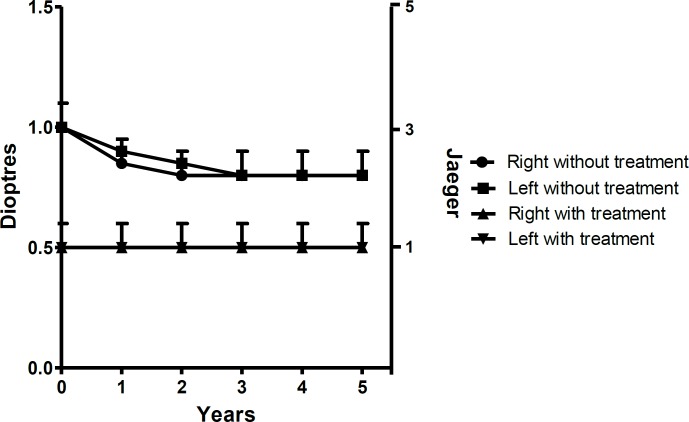
Near vision accommodation in patients in the pharmacological treatment. Accommodation in near vision in 200 emmetropic eyes of 100 patients treated with (▲ right eye) (▼ left eye) or without (without the drop for 24 hours) or with the combination of pilocarpine and diclofenac (● right eye) (■ left eye), during a 5 year period

As shown in Figure 1, in the first year of treatment, the enhanced accommodation improved near vision and this improved vision was maintained for 5 years. Distance vision remained at 20/20 and was unchanged during the same period. The combination of a muscarinic cholinergic agonist and NSAIDs allowed the chronic use of topical treatment to restore accommodation with good near and far vision.

## CONCLUSION

The restoration of accommodation can be achieved by stimulating ciliary muscle contractions with parasympathetic drug administration to modify the shape and position of the lens. The studied pharmacological treatment showed that the decrease in accommodation in presbyopic patients could be restored using a combination of pilocarpine with diclofenac. The possibility of this pharmacological treatment opens a new therapeutic approach for presbyopic patients, allowing them good accommodation over time. It is possible that, in the future, new pharmacological treatments can also be used to treat other refractive problems that depend on accommodation.

## DISCLOSURE

The authors report no conflicts of interest in this work.
